# Chickpeas from a Chilean Region Affected by a Climate-Related Catastrophe: Effects of Water Stress on Grain Yield and Flavonoid Composition

**DOI:** 10.3390/molecules27030691

**Published:** 2022-01-21

**Authors:** Adriano Costa de Camargo, Hernán Speisky, Raquel Bridi, Paula Núñez Pizarro, Arturo Larena, Ana Clara da C. Pinaffi-Langley, Fereidoon Shahidi, Andrés R. Schwember

**Affiliations:** 1 Laboratory of Antioxidants, Nutrition and Food Technology Institute, University of Chile, Santiago 7830490, Chile; hspeisky@inta.uchile.cl; 2 Departamento de Ciencias Vegetales, Facultad de Agronomía e Ingeniería Forestal, Pontificia Universidad Católica de Chile, Santiago 7820436, Chile; pjnunez@uc.cl; 3 Departamento de Química Farmacológica y Toxicológica, Facultad de Ciencias Químicas y Farmacéuticas, Universidad de Chile, Santiago 8380000, Chile; raquelbridi@ciq.uchile.cl; 4 Departamento de Farmacia, Facultad de Química, Pontificia Universidad Católica de Chile, Avda Vicuña Mackenna 4860, Santiago 7820436, Chile; alarena@uc.cl; 5 Department of Nutritional Sciences, College of Allied Health, University of Oklahoma Health Sciences Center, Oklahoma City, OK 73104, USA; anaclara-dacostapinaffilangley@ouhsc.edu; 6 Department of Biochemistry, Memorial University of Newfoundland, St. John’s, NL A1C 5ST, Canada; fshahidi@mun.ca

**Keywords:** *Cicer arietinum* L., drought stress, agricultural emergency, crop yield, phenolic compounds, biochanin A

## Abstract

The Valparaiso region in Chile was decreed a zone affected by catastrophe in 2019 as a consequence of one of the driest seasons of the last 50 years. In this study, three varieties (‘Alfa-INIA’, ‘California-INIA’, and one landrace, ‘Local Navidad’) of kabuli-type chickpea seeds produced in 2018 (control) and 2019 (climate-related catastrophe, hereafter named water stress) were evaluated for their grain yield. Furthermore, the flavonoid profile of both free and esterified phenolic extracts was determined using liquid chromatography-mass spectrometry, and the concentration of the main flavonoid, biochanin A, was determined using liquid chromatography with diode array detection. The grain yield was decreased by up to 25 times in 2019. The concentration of biochanin A was up to 3.2 times higher in samples from the second season (water stress). This study demonstrates that water stress induces biosynthesis of biochanin A. However, positive changes in the biochanin A concentration are overshadowed by negative changes in the grain yield. Therefore, water stress, which may be worsened by climate change in the upcoming years, may jeopardize both the production of chickpeas and the supply of biochanin A, a bioactive compound that can be used to produce dietary supplements and/or nutraceuticals.

## 1. Introduction

The International Year of Pulses 2016 highlighted the importance of pulses for human health, sustainability, and, most importantly, food security. In fact, a recent study [1] aiming to compare the frequency and quantity of different types of food consumed by adults from Santiago, Chile, before and during confinement, demonstrated that the consumption frequency of legume seeds (two to four times per week) in the capital of Chile increased during the COVID-19 pandemic year from 26% (pre-pandemic period) to 33.7% (lockdown period), thus confirming the critical role of legume seeds in food security. After dry beans, chickpea (*Cicer arietinum* L.) is the most important pulse [2].

Although chickpea is cultivated in more than 50 countries, India is the main producer and consumer, with a production that reaches almost 10 million tons, with approximately 60% of the global production [3]. Chile used to be a major producer and exporter of pulses, but this scenario has changed due to the emergence of more profitable crop alternatives, among other reasons. Some farmers have continued growing legumes for their subsistence, which is characterized by a low productive technological level. However, the harvested area increased 3.3 times from 2017 to 2019 [3], which may indicate a return to pulses production in the future.

From a nutritional perspective, chickpea is composed mainly of carbohydrates, but it is also an important source of protein (18.3–25.2%) and insoluble fiber (14.1–23.2%). In addition, chickpea has a lower lipid content than soybeans, thus representing a good addition to dietary interventions for weight and type II diabetes management. As for minor components, the seeds contain phenolic compounds, such as isoflavones. These secondary metabolites exert important functions in plants, including structural and protective (UV protection, antioxidant signaling, and defense against pathogens) functions. Furthermore, phenolic compounds, including isoflavones, provide a myriad of health benefits, which are, at least partially, attributed to their antioxidant and anti-inflammatory activity. Consequently, the consumption of chickpea has been associated with the prevention of cardiovascular diseases and certain types of cancer [4].

Biochanin A, the main isoflavone found in chickpea, is metabolized to form genistein through the action of 4′-*O*-methyltransferase as reported by Tolleson et al. [5] in a study using human liver microsomes. Therefore, some of the health benefits of chickpea consumption may be attributed to both biochanin A (at the gastrointestinal level) and genistein (at the systemic level). Besides chickpeas, other species of Fabaceae, such as soy (*Glycine max* (L.) Merr.), peanuts (*Arachis hypogaea* L.), Indian rosewood (*Dalbergia sissoo* Roxb. Ex DC.), golden tree (*Cassia fistula* L.), and alfalfa sprouts (*Medicago sativa* L.), are other natural sources of biochanin A [6]. Among pulses, some types of beans also contain biochanin A. However, chickpeas are by far the most important source of this bioactive compound according to data from the U.S. Department of Agriculture [7].

Regarding the potential of biochanin A as a multifunctional compound, Sarfraz et al. [6] highlighted its anti-inflammatory, neuroprotective, antioxidant, antimicrobial, hepatoprotective, and anticancer (specifically on cancers of the liver, prostate, breast, and pancreas) properties. According to the authors, biochanin A inhibits cellular growth and promotes cell apoptosis, thus exerting anti-tumorigenic effects. However, many of these properties remain to be adequately evaluated in humans [8]. In contrast, Nestel et al. [9] carried out a double-blinded trial to investigate the LDL-cholesterol-lowering effect of isoflavones in individuals who consumed a formulation enriched in biochanins. According to the same study, the consumption of this biochanin-rich supplement significantly decreased LDL-cholesterol levels only in men. In another study conducted by Clifton-Bligh et al. [10], postmenopausal women who consumed a commercially prepared isoflavone supplement containing genistein, daidzein, formononetin, and biochanin for six months showed an increase in HDL-cholesterol levels, a decrease in apolipoprotein B levels, and an increase in the cortical bone of the proximal radius and ulna.

Drought impacts plant growth and grain yield. With the progress of climate change in the last decades, this climate event has become a serious global food security issue [11]. Moreover, drought conditions affect the metabolism of plants [12]. Khan et al. [13] analyzed the metabolite accumulation of two chickpea varieties, one drought-resistant and another drought-sensitive variety, cultivated under drought stress conditions. They observed a considerable increase in the accumulation of tryptophan, L-arginine, L-histidine, L-isoleucine, L-proline, and allantoin. Farooq et al. [14] reported that polyols, organic acids, sugars, and amino acids are involved in the development of drought tolerance in plants in an intricate manner. More recently, Bhaskarla et al. [15] reported new insights into the drought tolerance mechanisms in roots that have important implications for chickpea improvement. However, studies focusing on the genetics and secondary metabolite production of chickpeas remain scarce [4].

Data from a meteorological station located 30 km north of the experimental site in the Valparaiso region of Chile revealed that the 2019 season had higher temperatures than the previous year, except for the months of July and September. The average highest temperatures recorded in 2018 and 2019 were 23.7 and 24.3 °C, respectively, whereas the lowest temperature recorded in these years was similar (3 °C). The annual precipitation for 2019 was 107 mm, with the highest precipitation recorded in June (70 mm). In July, precipitation decreased considerably, reaching values close to 0 mm in the following months. Comparably, the annual precipitation for 2018 was 209 mm, with rainfall progressively decreasing from June to October [16].

Considering that the Valparaiso region was decreed a zone affected by catastrophe in 2019 as a consequence of one of the driest seasons of the last 50 years [17], the importance of chickpea in the context of food security, and its potential as a feedstock to produce nutraceuticals and/or dietary supplements, the present study aimed to elucidate the effect of water stress on grain yield, flavonoid profile, and biochanin A concentration, the most abundant bioactive flavonoid of chickpea. For this purpose, we compared three varieties (‘Alfa-INIA’, ‘California-INIA’, and one landrace, ‘Local Navidad’) of kabuli-type chickpeas harvested in Chile before and after a climate-related catastrophe.

## 2. Results and Discussion

### 2.1. Grain Yield

The grain yield decreased by up to 25 times (19- to 25-fold lower yield in 2019), with the ‘Alfa-INIA’ variety the most affected by the water stress (Table 1). The yields were very low and below the national average of 3.2 qqm/Ha (0.21 ton/Ha) for the 2018/2019 season [18]. Furthermore, the national average yield was considered the lowest of the last 10 years. The effects of climate change and the increase in the world population require the development of chickpea varieties that can tolerate and thrive in drought conditions.

The abrupt decrease in yield may be a consequence of the severe drought that the central area of Chile has experienced in the last decade. This drought has mainly been caused by a reduction of prolonged rainfall, specifically in autumn and winter. In particular, 2019 was characterized as one of the driest years of the decade, mainly affecting the central and southern cultivation areas of the country. Consequently, Coquimbo and Valparaiso were declared zones affected by the catastrophe [17], with the latter being the location where the samples used in this study were grown.

### 2.2. Phenolic Profile

Daidzein, formononetin, genistein, biochanin A, luteolin, kaempferol, apigenin, isorhamnetin, and rutin were positively identified using liquid chromatography-mass spectroscopy (LC-MS/MS) and authentic standards. The instrumental parameters used for the LC–MS/MS analysis of the flavonoids examined are shown in Table 2 while the free and esterified flavonoids identified in chickpeas originating from a Chilean region affected by a climate-related catastrophe are reported in Table 3.

Mekky et al. [19] reported the presence of daidzein, biochanin A, genistein, kaempferol, apigenin, isorhamnetin, and rutin in chickpeas while Guardado-Félix et al. [20] reported the presence of formononetin. These authors also reported that the concentration of isoflavones from chickpeas increased from 118 to 3826 μg/g dw after four days of germination, which agrees with the results reported by earlier studies [21,22]. According to Fratianni et al. [23], luteolin was detected in crude extracts of the Castelcivita but not in those of the Sassano chickpea cultivar. The same compound was recovered from unfermented chickpeas. Moreover, while most studies (including those aforementioned) have only identified phenolic compounds present in crude extracts, we also evaluated the presence of phenolic compounds in free (Table 3) esterified (Table 4) forms, which is frequently ignored.

Overall, the chickpea cultivars differed slightly in their phenolic profile. Except for daidzen, all isoflavones were detected in the free fraction (Table 3) and in the one released from the esterified form (Table 4) in all samples of both seasons. ‘Local Navidad’ contained almost all investigated free flavonoids, expect isorhamnetin, whereas ‘California-INIA’ did not contain free luteolin and kaempferol. Furthermore, free kaempferol was not detected in ‘Alfa-INIA’. Regarding differences between free and esterified phenolic profiles, apigenin and isorhamnetin were not detected in the esterified fraction. Furthermore, while the diversity of free flavonoids appeared to decrease from season 1 (control) to season 2 (water stress), the diversity of esterified flavonoids increased or remained unchanged.

Regarding the concentration of flavonoids in chickpeas, biochanin A represented the most abundant compound, with its concentration reaching up to 95% of the total flavonoid content. Therefore, due to its representativeness, we chose biochanin A as a marker of water stress associated with chickpea-producing conditions. It is important to note that specific phenolics respond to drought conditions differently [24]. This is further illustrated by the differences between seasons shown in Table 3: daidzein, lutein, and isorhamnetin were not detected in season 1 (control) but were detected in season 2 (water stress), whereas kaempferol, apigenin, and rutin were detected in season 1 (control) but not detected in season 2 (water stress). Identifying these differences is important in terms of scientific knowledge. However, considering the minor contribution of these flavonoids compared with that of biochanin A, the impact of these changes on the extraction yield and, therefore, on the procurement of dietary supplements and/or nutraceuticals may be negligible.

**Figure 1:**
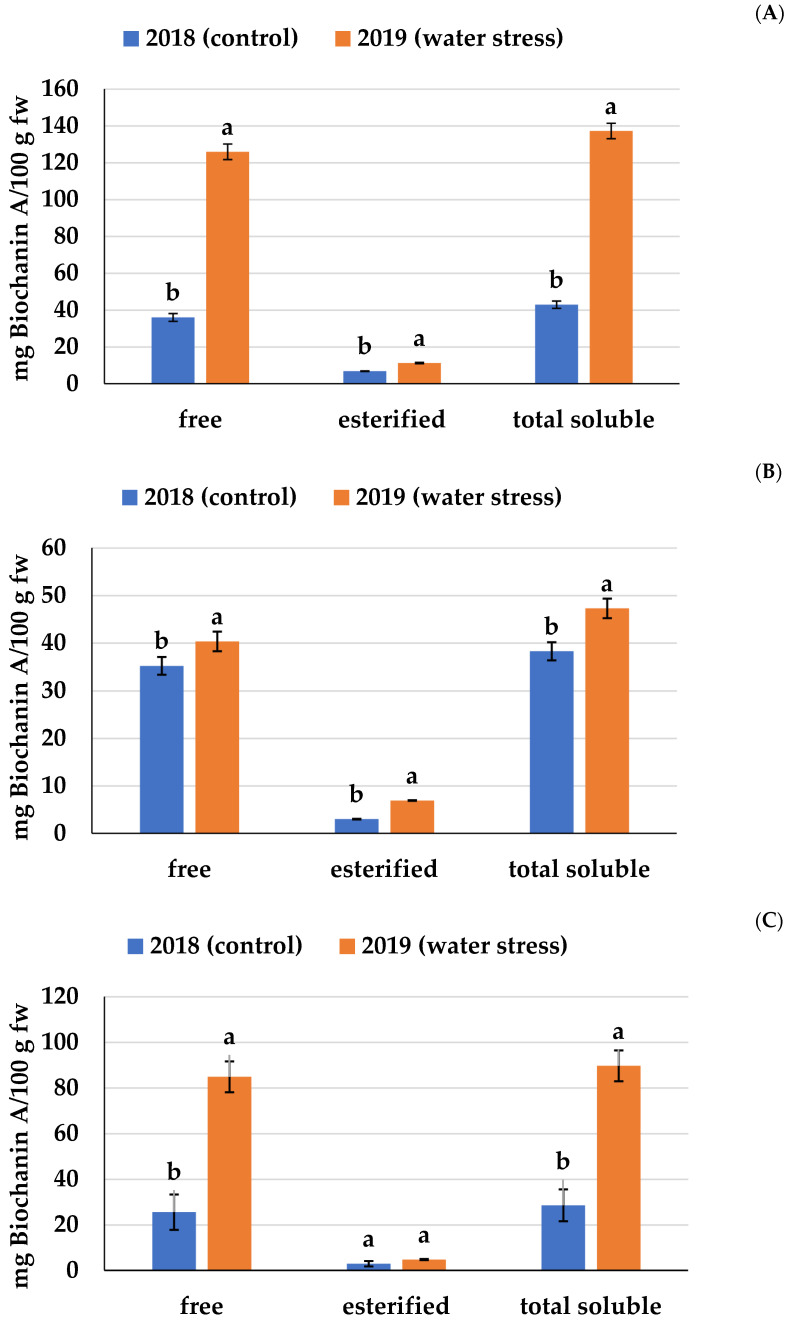
Effect of water stress on the concentration of biochanin A in chickpeas. ‘California-INIA’ (**A**), ‘Alfa-INIA’ (**B**), and ‘Local Navidad’ (**C**). a,b: Means with different letters within each fraction show differences between the control and water stress (*p* < 0.05).

Nonetheless, regardless of the season and variety, free biochanin A was the most prominent compound in chickpeas, ranging from 84 to 92% in the first season (control) and from 85% to 95% in the second season (water stress). Moreover, the concentration of esterified biochanin A (3–7 mg/100 fw g of sample (season 1, control) and 5–11 mg/100 g fw (season 2, water stress)) is not negligible (Figure 1). In fact, this surpasses the content found in other common sources of biochanin A, such as sprouted alfalfa seeds (0.04 mg/100 g fw), apricots (0.05 mg/100 g fw), different types of raw or cooked beans (0.04–0.60 mg/100 g fw), clover sprouts (0.59 mg/100 g fw), cowpeas (0.58 mg/100 g fw), and pigeon peas (0.10 mg/100 g fw) [25].

Thus, ignoring the presence of esterified biochanin A may lead to an underestimation of the real concentration of this flavonoid in the starting material. The total content of biochanin A (free + esterified), which ranged from 28.6 mg/100 g (‘Alfa-INIA’ Season 1, control) to 137.3 mg/100 g (‘California-INIA’ Season 2, water stress), agreed with that reported by previous studies (18–200 mg/100 g) [21,26]. In summary, the present study improves the knowledge about natural sources of biochanin A by comparing its concentration in chickpeas with that in other common sources and by demonstrating that a significant part of this isoflavone is present in an esterified form.

To the best of our knowledge, there are no published studies on the effects of severe water stress on the biochanin A concentration in chickpeas. The concentration of total biochanin A (Figure 1) was up to 3.2 times higher in the second season (water stress) than in the first season (water stress). This overall increase reflected the increment in both the free (up to 3.5-fold) and esterified (up to 2.3-fold) biochanin A concentrations. Finally, our results suggest that water stress preferentially modulates the biosynthesis of free rather than esterified biochanin A. This possibility merits further investigation.

Our results are supported by literature data on the isoflavones content of common beans subjected to restricted irrigation and severe drought [24]. According to Herrera et al. [24], the concentration of 6′′-*O*-malonylgenistin, the main isoflavone present in common beans, increased by 84% in samples subjected to severe drought. In the present study, although a greater content of biochanin A was found in samples from the drought season, such positive changes in the phenolic composition were overshadowed by the negative change in the grain yield.

As mentioned in a previous report [4], studies focusing on the genetics and secondary metabolite production of chickpeas remain scarce. A recent transcriptomic study using wild and domesticated chickpea grown under water stress revealed metabolic pathways (e.g., phenylpropanoid metabolism) and biological processes (e.g., stomatal development) that are differentially controlled between the wild and domesticated varieties, with potential consequences for drought tolerance. In this sense, wild chickpea may be useful for breeding plants that are tolerant of drought conditions [27]. Further studies investigating the genes encoding for enzymes involved in the phenylpropanoid pathway and their response under severe drought stress are needed. This would be helpful to better understand the changes in the flavonoid composition of chickpeas subjected to water stress.

## 3. Materials and Methods

### 3.1. Experimental Design

Three varieties (‘Alfa-INIA’, ‘California-INIA’, and one landrace, ‘Local Navidad’) of Kabuli-type chickpea seeds cultivated in 2018 (control) and 2019 (drought-related catastrophe) were evaluated. The varieties were grown in the Valparaíso region of Chile (33°51′32″ S, 71°46′3″ W; altitude: 26 m). Randomized blocks in a split-split-plot arrangement were sown in October 2018 and September 2019. The 4-m^2^ plots comprised 3 equally spaced rows in which 14 seeds per linear meter were planted (7 cm apart from one another). Overall, a density of 25 seeds/m^2^ was achieved to obtain 20 plants/m^2^.

### 3.2. Chemical Compounds

Daidzein, formononetin, genistein, biochanin A, luteolin, kaempferol, apigenin, isorhamnetin, and rutin were purchased from Sigma-Aldrich (St. Louis, MO, USA).

### 3.3. Extraction of Phenolic Compounds

Dry chickpea samples were portioned, added to water (1:3 w:v), and macerated for 15 h at 5 °C [28]. Although other methods, such as seed milling [29], exist, maceration is commonly used because it decreases the concentration of antinutritional oligosaccharides, such as raffinose and stachyose [30]. Thus, were opted to employ maceration in our study [28]. After draining the water, the chickpeas were mixed with a solution of methanol–acetone–water (7:7:6 v:v:v) [31] and homogenized for 2 min [28] using a blender (Oster, Model BRLY07-Z00, Milwaukee, WI, USA). The homogenized samples were subsequently centrifuged at 4000× *g* for 5 min (Z-326 K, Hermle Labortechnik GmbH, German). The supernatant was collected, and the extraction cycle was repeated twice. The organic solvent was removed using evaporation. The remaining aqueous solution was acidified (pH 2) using hydrochloric acid (6 M). Free phenolic compounds were extracted from this acidified solution using diethyl ether–ethyl acetate (1:1, v:v; total extraction cycles: 5). The organic phase was collected, and the sample was dried under vacuum at 40 °C (mLab scientific HCM 100-pro). The remaining water phase was alkalinized using sodium hydroxide (4 M; 1:1 v:v), and a basic hydrolysis reaction was carried out under constant stirring and an inert atmosphere (N_2_) for 4 h at 23–25 °C to promote the release of soluble esterified phenolic compounds. Subsequently, the mixture was acidified to pH 2 using hydrochloric acid (6 M) and the released compounds were extracted as described for the free phenolic compounds [31,32,33,34,35,36]. The free and esterified phenolic fractions were reconstituted in HPLC-grade methanol and stored at −80 °C until further analysis.

### 3.4. UPLC-MS/MS Analysis

Flavonoids were identified using an ABSciex triple Quad 4500 mass spectrometer equipped with an electrospray (TurboV) interface coupled to an Eksigent Ekspert Ultra LC100 with an Ekspert Ultra LC100-XL autosampler system (AB/Sciex Concord, ON, Canada). Chromatographic separation occurred using a gradient elution with (A) 0.1% formic acid and (B) methanol as the mobile phase as follows: 0–1 min, 15% B; 1–17 min, 15–100% B; 17–21 min 100–100% B; 21–22 min, 100–15% B; and 22–25 min, 15–15% B. The instrument was operated using an injection volume of 50 μL, a flow rate of 0.5 mL/min, and an end-capped column (LiChrospher 100 RP-18; 125 mm × 4 mm i.d., 5 μm; Merck, Darmstadt, Germany) maintained at 50 °C. Calibration curves for quantification were constructed using commercially available standards. Table 1 shows the parameters used for compound identification.

### 3.5. HPLC-DAD Analysis

Free and esterified biochanin A were analyzed using a Hitachi Chromaster 5000 series high-performance liquid chromatographer equipped with an autosampler, a photodiode array detector, and a Chromaster system manager V1.2 (Hitachi, Tokyo, Japan). Chromatographic separation was carried out using a gradient elution with (A) methanol, (B) acetonitrile, and (C) 0.1% formic acid as the mobile phase as follows: 0–10 min: 20% B and 80% C; 10.1–40 min: 7.5% A, 25% B, and 67.5% C; 40.1–50 min: 15% A, 25% B, and 60% C; 50.1–65 min: 15% A, 45% B, and 40% C; 65.1–80 min: 20% B and 80% C. The instrument was operated using an injection volume of 10 µL, a flow rate of 0.8 mL/min, an end-capped column (Purospher STAR RP-18; 250 mm × 4.6 mm i.d.; same type of guard column), and an oven column temperature of 35 °C. The absorbance was monitored in the wavelength range of 210–550 nm and the chromatograms were integrated at 260 nm. Calibration curves for quantification were constructed using a commercially available standard for biochanin A.

### 3.6. Statistical Analysis

All analyses were performed in triplicate. Results were compared for differences using analysis of variance and Tukey’s test. The statistical significance level was set at α = 5%. All analyses were conducted using SAS software (SAS Institute, Cary, NC, USA).

## 4. Conclusions

The effects of climate change become more apparent each year. Among these, changes in weather patterns, such as insufficient rainfall, endanger the production of pulses and may result in increased food insecurity, as exemplified by the drastically low grain yield observed in this study with chickpeas cultivated in a drought-stricken region. Thus, besides efforts directed at mitigating the effects of climate change, we must also direct efforts at developing cultivars resistant to extreme weather conditions. Furthermore, the effects of environmental stress on the biosynthesis of bioactive compounds, such as biochanin A, must be further investigated.

## Figures and Tables

**Table 1:** Grain yield (ton/ha) of chickpea cultivars harvested in 2018 (control) and 2019 (water stress).

Sample	‘California-INIA’	‘Alfa-INIA’	‘Local Navidad’
Control	2.39 ± 0.02 a	2.47 ± 0.48 a	2.75 ± 0.17 a
Water stress	0.12 ± 0.01 b	0.10 ± 0.01 b	0.14 ± 0.02 b

Control: 2018; Water stress: 2019. Results are expressed as mean (*n* = 3) ± standard deviation. Results followed by the same lowercase letter in the column do not differ significantly (Turkey test, *p* < 0.05).

**Table 2:** Parameters used for the LC–MS/MS analysis of the examined flavonoids.

Compound	MRM Transition 1	DP	CE	CXP	MRM Transition 2	DP	CE	CXP
Daidzein	252.9 > 131.7	−105	−50	−9	252.9 > 207.7	−105	−44	−1
Formononetin	267.1 > 251.6	−110	−26	−9	267.1 > 222.9	−110	−46	−9
Genistein	268.8 > 133.0	−170	−38	−43	268.8 > 181.0	−170	−34	−13
Biochanin A	282.9 > 267.9	−80	−32	−5	282.9 > 211.1	−80	−46	−5
Luteolin	285.0 > 133.0	−125	−42	−5	285.0 > 150.9	−125	−34	−11
Kaempferol	285.0 > 184.9	−135	−36	−15	285.0 > 116.9	−135	−48	−3
Apigenin	268.9 > 117.0	−130	−40	−9	268.9 > 150.9	−130	−32	−5
Isorhamnetin	315.0 > 299.9	−130	−32	−15	315.0 > 150.9	−130	−40	−11
Rutin	609.0 > 299.8	−170	−50	−13	609.0 > 300.5	−170	−42	−9

MRM: multiple reaction monitoring; DP: declustering potential; CE: collision energy; CXP: collision cell exit potential.

**Table 3:** Free flavonoids identified in chickpeas originating from a Chilean region affected by a climate-related catastrophe.

Free Flavonoids	‘California-INIA’		‘Alfa-INIA’		‘Local Navidad’	
	Control	Water Stress	Control	Water Stress	Control	Water Stress
Daidzein	(+)	(+)	(+)	(+)	(+)	(+)
Formononetin	(+)	(+)	(+)	(+)	(+)	(+)
Genistein	(+)	(+)	(+)	(+)	(+)	(+)
Biochanin A	(+)	(+)	(+)	(+)	(+)	(+)
Luteolin	nd	nd	(+)	(+)	(+)	(+)
Kaempferol	nd	nd	nd	nd	(+)	nd
Apigenin	(+)	nd	(+)	(+)	(+)	nd
Isorhamnetin	(+)	(+)	(+)	(+)	nd	(+)
Rutin	(+)	nd	(+)	(+)	(+)	(+)

Control: 2018; Water Stress: 2019. (+), detected; nd, not detected.

**Table 4:** Esterified flavonoids identified in chickpeas originating from a Chilean region affected by a climate-related catastrophe.

Esterified Flavonoids	‘California-INIA’		‘Alfa-INIA’		‘Local Navidad’	
	Control	Water Stress	Control	Water Stress	Control	Water Stress
Daidzein	nd	(+)	nd	nd	nd	nd
Formononetin	(+)	(+)	(+)	(+)	(+)	(+)
Genistein	(+)	(+)	(+)	(+)	(+)	(+)
Biochanin A	(+)	(+)	(+)	(+)	(+)	(+)
Luteolin	nd	nd	nd	(+)	(+)	(+)
Kaempferol	(+)	(+)	nd	nd	(+)	(+)
Apigenin	nd	nd	nd	nd	nd	nd
Isorhamnetin	nd	nd	nd	nd	nd	nd
Rutin	(+)	(+)	(+)	(+)	(+)	(+)

Control: 2018; Water Stress: 2019. (+), detected; nd, not detected.

## Data Availability

The data presented in this study are available on request from the first author.

## References

[1] Pye C., Sutherland S., Martín P.S. (2021). Consumo de frutas, verduras y legumbres en adultos de Santiago Oriente, Chile: ¿Ha influido el confinamiento por COVID-19?. Revista Chilena Nutrición.

[2] Rawal V., Navarro D.K. (2019). The Global Economy of Pulses.

[3] FAOSTAT Food and Agriculture Organization of the United Nations. https://www.fao.org/faostat/en/.

[4] de Camargo C.A., Favero T.B., Morzelle C.M., Franchin M., Alvarez-Parrilla E., de la Rosa A.L., Geraldi V.M., Maróstica Júnior R.M., Shahidi F., Schwember R.A. (2019). Is chickpea a potential substitute for soybean? Phenolic bioactives and potential health benefits. Int. J. Mol. Sci..

[5] Tolleson W.H., Doerge D.R., Churchwell M.I., Marques M.M., Roberts D.W. (2002). Metabolism of biochanin A and formononetin by human liver microsomes in vitro. J. Agric. Food Chem..

[6] Sarfraz A., Javeed M., Shah M.A., Hussain G., Shafiq N., Sarfraz I., Riaz A., Sadiqa A., Zara R., Zafar S. (2020). Biochanin A: A novel bioactive multifunctional compound from nature. Sci. Total Environ..

[7] U.S. Department of Agriculture, Agricultural Research Service (2015). USDA Database for the Isoflavone Content of Selected Foods, Release 2.1. Nutrient Data Laboratory Home Page. U.S. Department of Agriculture. https://data.nal.usda.gov/dataset/usda-database-isoflavone-content-selected-foods-release-20.

[8] Yu C., Zhang P., Lou L.X., Wang Y. (2019). Perspectives Regarding the Role of Biochanin A in Humans. Front. Pharmacol..

[9] Nestel P., Cehun M., Chronopoulos A., DaSilva L., Teede H., McGrath B. (2004). A biochanin-enriched isoflavone from red clover lowers LDL cholesterol in men. Eur. J. Clin. Nutr..

[10] Clifton-Bligh P.B., Baber R.J., Fulcher G.R., Nery M.L., Moreton T. (2001). The effect of isoflavones extracted from red clover (Rimostil) on lipid and bone metabolism. Menopause.

[11] Lobell D.B., Roberts M.J., Schlenker W., Braun N., Little B.B., Rejesus R.M., Hammer G.L. (2014). Greater sensitivity to drought accompanies maize yield increase in the US Midwest. Science.

[12] Witt S., Galicia L., Lisec J., Cairns J., Tiessen A., Araus J.L., Palacios-Rojas N., Fernie A.R. (2021). Metabolic and phenotypic responses of greenhouse-grown maize hybrids to experimentally controlled drought stress. Mol. Plant.

[13] Khan N., Bano A., Rahman M.A., Rathinasabapathi B., Babar M.A. (2018). UPLC-HRMS-based untargeted metabolic profiling reveals changes in chickpea (*Cicer arietinum*) metabolome following long-term drought stress. Plant Cell Environ..

[14] Farooq M., Hussain M., Wahid A., Siddique K.H.M., Aroca R. (2012). Drought stress in plants: An overview. Plant Responses to Drought Stress.

[15] Bhaskarla V., Zinta G., Ford R., Jain M., Varshney R.K., Mantri N. (2020). Comparative root transcriptomics provide insights into drought adaptation strategies in chickpea (*Cicer arietinum* L.). Int. J. Mol. Sci..

[16] DGAC Dirección Meteorológica De Chile, Contigo Todo El Tiempo. https://www.dgac.gob.cl/direccion-meteorologica-de-chile-contigo-todo-el-tiempo/.

[17] BCN Decreto 308—Declara Como Zona Afectada por Catástrofe a las Comunas de las Regiones de Coquimbo y Valparaíso que Indica. https://www.bcn.cl/leychile/navegar?idNorma=1136546.

[18] ODEPA Estadísticas Productivas. https://www.odepa.gob.cl/estadisticas-del-sector/estadisticas-productivas.

[19] Mekky R.H., Contreras M.D., El-Gindi M.R., Abdel-Monem A.R., Abdel-Sattar E., Segura-Carretero A. (2015). Profiling of phenolic and other compounds from Egyptian cultivars of chickpea (*Cicer arietinum* L.) and antioxidant activity: A comparative study. RSC Adv..

[20] Guardado-Felix D., Serna-Saldivar S.O., Cuevas-Rodriguez E.O., Jacobo-Velazquez D.A., Gutierrez-Uribe J.A. (2017). Effect of sodium selenite on isoflavonoid contents and antioxidant capacity of chickpea (*Cicer arietinum* L.) sprouts. Food Chem..

[21] Gao Y., Yao Y., Zhu Y.Y., Ren G.X. (2015). Isoflavone Content and Composition in Chickpea (*Cicer arietinum* L.) Sprouts Germinated under Different Conditions. J. Agric. Food Chem..

[22] Wu Z., Song L., Feng S., Liu Y., He G., Yioe Y., Liu S.Q., Huang D. (2012). Germination Dramatically Increases Isoflavonoid Content and Diversity in Chickpea (*Cicer arietinum* L.) Seeds. J. Agric. Food Chem..

[23] Fratianni F., Cardinale F., Cozzolino A., Granese T., Albanese D., Di Matteo M., Zaccardelli M., Coppola R., Nazzaro F. (2014). Polyphenol composition and antioxidant activity of different grass pea (*Lathyrus sativus*), lentils (*Lens culinaris*), and chickpea (*Cicer arietinum*) ecotypes of the Campania region (Southern Italy). J. Funct. Foods.

[24] Herrera M.D., Acosta-Gallegos J.A., Reynoso-Camacho R., Perez-Ramirez I.F. (2019). Common bean seeds from plants subjected to severe drought, restricted- and full-irrigation regimes show differential phytochemical fingerprint. Food Chem..

[25] Raheja S., Girdhar A., Lather V., Pandita D. (2018). Biochanin A: A phytoestrogen with therapeutic potential. Trends Food Sci. Technol..

[26] Zhang L., Li Q., Yang X.D., Xia Z.L. (2012). Effects of Sodium Selenite and Germination on the Sprouting of Chickpeas (*Cicer arietinum* L.) and Its Content of Selenium, Formononetin and Biochanin A in the Sprouts. Biol. Trace Elem. Res..

[27] Moenga S.M., Gai Y., Carrasquilla-Garcia N., Perilla-Henao L.M., Cook D.R. (2020). Gene co-expression analysis reveals transcriptome divergence between wild and cultivated chickpea under drought stress. Plant J..

[28] Silva M.B.R., Falcão H.G., Kurozawa L.E., Prudencio S.H., de Camargo A.C., Shahidi F., Ida E.I. (2019). Ultrasound- and hemicellulase-assisted extraction increase β-glucosidase activity, the content of isoflavone aglycones and antioxidant potential of soymilk. J. Food. Bioact..

[29] Johnson J.B., Walsh K.B., Bhattarai S.P., Naiker M. (2021). Partitioning of nutritional and bioactive compounds between the kernel, hull and husk of five new chickpea genotypes grown in Australia. Future Foods.

[30] Han I.H., Baik B. (2006). Oligosaccharide Content and Composition of Legumes and Their Reduction by Soaking, Cooking, Ultrasound, and High Hydrostatic Pressure. Cereal Chem..

[31] Ayoub M., De Camargo A.C., Shahidi F. (2016). Antioxidants and bioactivities of free, esterified and insoluble-bound phenolics from berry seed meals. Food Chem..

[32] De Camargo A.C., Regitano-d’Arce M.A.B., Gallo C.R., Shahidi F. (2015). Gamma-irradiation induced changes in microbiological status, phenolic profile and antioxidant activity of peanut skin. J. Funct. Foods.

[33] Ambigaipalan P., de Camargo A.C., Shahidi F. (2016). Phenolic Compounds of Pomegranate Byproducts (Outer Skin, Mesocarp, Divider Membrane) and Their Antioxidant Activities. J. Agric. Food Chem..

[34] Rahman M.J., de Camargo A.C., Shahidi F. (2018). Phenolic profiles and antioxidant activity of defatted camelina and sophia seeds. Food Chem..

[35] Albishi T., Banoub J.H., de Camargo A.C., Shahidi F. (2019). Wood extracts as unique sources of soluble and insoluble-bound phenolics: Reducing power, metal chelation and inhibition of oxidation of human LDL-cholesterol and DNA strand scission. J. Food Bioact..

[36] Mudenuti N.V.R., de Camargo A.C., de Alencar S.M., Danielski R., Shahidi F., Madeira T.B., Hirooka E.Y., Spinosa W.A., Grossmann M.V.E. (2021). Phenolics and alkaloids of raw cocoa nibs and husk: The role of soluble and insoluble-bound antioxidants. Food Biosci..

